# The West Africa Field Epidemiology and Laboratory Training Program, a strategy to improve disease surveillance and epidemic control in West Africa

**Published:** 2011-12-14

**Authors:** Evariste Mutabaruka, Mamadou Sawadogo, Zekiba Tarnagda, Lauren Ouédraogo, Lassana Sangare, Badolo Ousmane, Yassa Ndjakani, Olivia Namusisi, David Mukanga, Michele Evering-Watley, Sennen Hounton, Peter Nsubuga

**Affiliations:** 1 World Health Organization/Multi-Disease Surveillance Center, Ouagadougou, Burkina Faso; 2 University of Ouagadougou, Burkina Faso; 3 African Field Epidemiology Network, Kampala, Uganda; 4 Centers for Global Health, Center for Disease Control and Prevention, Atlanta Georgia, USA

**Keywords:** Epidemiology, West Africa, field epidemiology and laboratory training program, surveillance

## Abstract

The West Africa Field Epidemiology and Laboratory Training Program (WA-FELTP) which was established in September 2007, is an inter-country, competency-based, in-service and post -graduate training program in applied epidemiology and public health that builds the capacity to strengthen the surveillance and response system as well as epidemic control in the French-speaking countries where they are implemented. The overall purpose is to provide epidemiological and public health laboratory services to the public health systems at national, provincial, district and local levels. The program includes four countries: Burkina Faso, Mali, Niger, and Togo with an overarching goal to progressively cover all French speaking countries in West Africa through a phased-in approach. WA-FELTP's 2- year Master's program was launched in 2010 with 12 residents, three from each country, and consists of medical and veterinary doctors, pharmacists, and laboratory scientists. The training comprises 25% didactic sessions and 75% practical in-the-field mentored training. During the practical training, residents rovide service to their respective ministries of health and ministries of animal resources by contributing to outbreak investigations and activities that help to improve national surveillance systems at national, regional, district and local levels. The pressing challenges that the program must address consist of the lack of funds to support the second cohort of trainees, though trainee selection was completed, inadequate funds to support staff compensation, and shortage of funds to support trainees’ participation in critical activities in field epidemiology practice, and a need to develop a 5-year plan for sustainability.

## Introduction

he West Africa Field Epidemiology and Laboratory Training Program (WA-FELTP) which was established in September 2007, is an inter-country, competency-based, in-service and post -graduate training program in applied epidemiology and public health that aims at building capacity to strengthen the public health surveillance and response system as well as epidemic control in West African French-speaking countries. The overall goal of Field Epidemiology Training Programs is to enhance public health capacity by developing a cadre of health professionals with advanced skills in applied epidemiology and laboratory management and to provide epidemiological and biological services to the public health systems at national, provincial, district and local levels. The program includes four countries: Burkina Faso, Mali, Niger and Togo with an overarching goal to progressively cover all French-speaking countries in West Africa [1]. The WA-FELTP is a partnership between ministries of health, ministries of higher education, ministries of animal resources, and leading universities, research institutions, and reference laboratories in the four countries. Other partners are the World Health Organization (WHO)'s Multi Disease Surveillance Centre (MDSC), the United States (US) Centers for Disease Control and Prevention (CDC), the US Agency for International Development (USAID), the West Africa Health Organization (WAHO), and the African Field Epidemiology Network (AFENET). The WA-FELTP is housed at MDSC and the University of Ouagadougou is the host institute for the Master FELTP, and is also the awarding entity for degrees attained by trainees of the program in academic agreement with the other concerned universities (as an accredited university of the Higher Education Council for French speaking countries-CAMES) [2].

The governing and advisory bodies of the WA-FELTP are: the Steering Committee, the Program Director at MDSC, the Host University Master FELTP Director, and the Scientific Advisory Committee. The focal persons of WA-FELTP in each country are the four Directors of Prevention and Disease Control within the ministries of health of the participating country, and representatives from involved universities, research institutes and references laboratories.

### Vision and mission

The program envisions growing into a leading professional training program that advances public health in West Africa and beyond, by addressing their public health needs and priorities through training and service in applied epidemiology and laboratory management. The program goes beyond training by working with ministries of health and ministries of animal resources in building a sustainable network of highly skilled field epidemiologists and laboratory managers, as well as a roster of frontline health worker field staff who are measurably improving public health services through: a) successful and timely outbreak investigation and response capacity, b)creating functional public health surveillance systems, c)developing functional laboratory capacity, d)evidence-based decision making, enhanced collaboration, networking and research.

## Objectives

The objectives of the program are to strengthen the capacity public health workers in Francophone West Africa to improve national public health surveillance systems and thereby providing timely response to outbreaks and other public health emergencies. The program also aims to enhance public health capacity by developing a cadre of health professionals with advanced skills in applied epidemiology and laboratory management and to provide public health services at national and sub-national levels.

### Description of the Program

The 2-year FELTP program was launched in 2010 with the first cohort of 12 residents comprising three from each participating country, and consisting of medical and veterinary doctors, and medical biologists and a pharmacist from Burkina Faso. WA-FELTP's philosophy is to have epidemiologists and medical biologists learn about the role of the various public health professionals in controlling public health problems. The training comprises 25% didactic sessions and 75% practical in-the-field mentored training. During the practical training, residents provide service to their respective ministries of health and ministries of animal resources by contributing to outbreak investigations and activities that help to improve national surveillance systems at national, provincial, district and local levels. Upon completion residents will receive a Master of Public Health (MPH) degree in Applied Epidemiology (and Laboratory Management for the laboratory scientists) and will be posted to serve in key public health and animal health programs to advance good public health practices in the region [2]. To facilitate MPH degree and short course trainings, the WA–FELTP uses experienced international professors and trainers from the four concerned universities, public health and research institutes, and reference laboratories (including the School of Veterinary Medicine in Dakar, Senegal, the Pasteur Institute of Dakar, Senegal), WHO, and CDC.

Additionally, WA-FELTP conducts a series of short course trainings for public health workers in the participating countries. The short courses provide training in outbreak investigations and the use of evidence-based decision-making in public health. During each short course participants receive a project assignment and they report their findings after 3 months. The project presentations are evaluated by panel of judges who judge the presenters’ quality of thought, practicality, and ability to address the problem itself. The overall objective of these courses is to help strengthen the capacity of countries to plan, implement, monitor and evaluate public health surveillance systems for priority diseases (e.g., cerebral spinal meningitis) in the West African Region. Since 2007, the program has trained a total of 121 health professionals through short course trainings conducted from the four WA-FELTP countries. Through its short course trainings, the WA –FELTP is progressively building a roster of skilled health field staff in outbreak investigation and response within the target countries who will contribute to a critical mass of health personnel to be deployed anytime to fight recurrent epidemics in Francophone West Africa [3].WA-FELTP is an excellent example where the “One Health” concept is demonstrated through joint human-animal health training and service in disease surveillance, outbreak investigation and response, epidemiological studies and public health management/leadership that address priority public health challenges in African countries [4,5].

WA-FELTP offers a pathway to achieve a major goal of the World Health Organization for Regional Office for Africa's Integrated Disease Surveillance and Response (IDSR) strategy, which is to strengthen district-level surveillance capacities for detecting, confirming and responding to priority diseases that afflict African communities, and linking public health surveillance with laboratory support in order to produce relevant and high-quality information for taking public health action [6].

## Achievements and highlights of the program

The key achievements of the WA-FELTP are listed in Table 1 and include:. Adoption of the WA-FELTP program as a degree of University of Ouagadougou; Validation and adoption of the Master FELTP curriculum by the University of Ouagadougou Scientific Committee; Ministry of Education decree to recognize the Master FELTP as the official degree for public health servants in Burkina Faso (and hence for French speaking countries).

**Table 1:** Key Achievements of the West Africa Field Epidemiology and Laboratory Training Program

Achievements	Number
Outbreaks investigations and response	5
Surveillance data publication	6
Surveillance systems evaluated	4
Research studies done	12 (thesis)
Evaluations (Program or Project)	2
Scientific presentations at conferences	2
Publications by the trainees	–
Abstracts for AFENET, EIS, and TEPHINET	22
Public health workers trained in short courses	121
Public health projects conducted by short course trainees	91

### Outbreak response activities

1) Investigation of Cholera outbreaks in Lome, Togo (May 2011) and Niger (September 2010 and August 2011); 2) Investigation of Measles outbreak in Bamako, Mali (March 2010); 3) Meningitis Vaccination campaigns organized by WHO in Burkina Faso (December 2010); 4) Investigation of Yellow fever case in Bobo, Burkina Faso (November 2010); 5) Investigation of Meningitis outbreak in Barsalogho, Burkina Faso (March 2010)

### Surveillance systems and evaluation

1) An evaluation of the measles surveillance system in Mali. Recommendations to the Ministry of Health were formulated to improve data collection and reporting, training of health workers, and the need of increasing the staff; 2) Epidemiological surveillance of contagious bovine pleura -pneumonia in Mali. Residents demonstrated the strengths and weaknesses of the surveillance system, and made some recommendations to the Animal Resources Department for improvement; 3) Epidemiological surveillance system for meningitis in Kolokani, Mali, May 2010; 4) Surveillance of highly pathogenic H5N1 avian influenza in Burkina Faso-2010; 5) An evaluation of the filariasis campaign in Burkina Faso by two residents of Burkina Faso who found out that filariasis is still a problem despite distribution of ivermectinin the country, and recommendations made to the Burkina Faso Ministry of Health; 6) An evaluation of meningitis surveillance data from 2009– 2010 in Burkina Faso; 7) An evaluation and organization of electronic database system for the district of Kati in Mali. The residents set up a system which will be used for IDSR implementation, including the Expanded Program for Immunization and outbreak investigations; 8) An evaluation of the surveillance of human and animal rabies in two districts of Togo. The surveillance findings indicated that rabies is still a problem in Togo, and children are the most affected. Also, there is not enough anti-rabies vaccine in Togo, and the reporting system need to be improved as recommendation was formulated by the residents to the official of animal resources; 9) Evaluation of Malaria in Central –East Sanitary Region, Burkina Faso, 2006–2009; 10) Evaluation of Meningitis’ Surveillance System –Ziniare –Burkina Faso, 2003–2010

### Short Courses

The program has trained a total of 121 health professionals through short course trainings conducted from 2007– 2011 as listed below.

1) Outbreak Investigation Short Course in Burkina Faso in 2007 and 2008. Sixty-one participants were trained, including district and regional Medical Officers, Surveillance Officers, Biologists and Pharmacists. The training was followed by presentations of mini projects implemented by participants three months after the short course training occurred; 2) Outbreak Investigation Short Course in Mali in 2008. Twenty district and regional medical officers, surveillance officers, biologists and pharmacists were trained. This was followed by presentations of mini projects after a three month period; 3) Outbreak Investigation Short Course in Lome, Togo, June 2010. Twenty district and regional medical officers, surveillance officers, biologists, pharmacists and veterinarians were trained; 4) Regional Workshop on Pandemic Influenza Preparedness and Response in Central and West Africa was held in Ouagadougou, Burkina Faso in July 2011. A total of 27 participants from eight countries attended including: FELTP residents, ministries of health and animal personnel. The training focused on key aspects of preparedness and responses to pandemic influenza. Participants of each country identified a mini project topic, and the project will be implemented and reports submitted by the end of 2011.

### Sustainability

The initial successes of the WA-FELTP have been possible so far due to seed funding provided by USAID and CDC and contributions in various forms by the University of Ouagadougou and other partners, including the ministries of health of the four initial countries. Further achievement and support will largely depend on a widened partner base as well as continued funding from respective ministries of health to support future residents from their country to participate in the applied epidemiology program. Although the regional WA-FELTP began with four member countries in Francophone Africa, the desire, once the program is well established and has identified and secured additional funding, is to expand the program to include other Francophone countries throughout Africa through a phased in approach.

## Challenges

Despite the initial successes noted, above WA-FELTP is experiencing difficulties particularly in obtaining funding to support the 2-year Master's FELTP beyond the first cohort. This shortfall of funding can be attributed to the prevailing economic difficulties in the global economy and the difficulty in obtaining a wider donor base for the program despite several attempts. However, countries and partners, as well as the host university (University of Ouagadougou) have shown dedicated support for short courses and the Master's program and this support needs to be translated into support for the 2-year Master's FELTP. There is a need for a 5-year plan that will allow a phasing approach to demonstrate program added value and to achieve complete support by governments.

## Conclusion

There is an unmet need for field epidemiologists and public health lab in the West African sub-region. The WA-FELTP program helps to bridge the gaps in the realm of field epidemiology training and the first program of its nature in Francophone Africa. WAFELTP is ensuring that trainees receive intense training on appropriate responses to public health emergencies. The outbreak response and surveillance system evaluation, along with over 100 short course trainees demonstrates that this type of training and program is viable in Francophone West Africa, an area which has a great need for this training and those efforts from national governments and other donors need to be intensified to continue the program. The graduates of this program, like alumni of other FELTPs will play a central role in public health surveillance, disease control, implementation and evaluation of public health programmes (e.g., in malaria, tuberculosis, HIV/AIDS, maternal and child health immunisation program), and in outbreak investigation and control. FELTP graduates have the potential to be in leadership positions in ministries of health, non-governmental organizations, and other health agencies. They also have implemented cross-border public health surveillance systems that have contributed significantly to reducing transmission of diseases and promoted enforcement of the International Health Regulations (1&6). In the future, the WA-FELTP is planning to extend the program to other Francophone African countries that are not currently benefiting from participating in the WA-FELTP, and creating an alumni association and scientific journal for submitting articles, and sharing findings. Sustaining the early successes of WA-FELTP will depend on continuation of funding from all the stakeholders.
